# Facteurs associés à la grossesse post-terme au Centre Hospitalier Universitaire Yalgado Ouédraogo: étude cas-témoins

**DOI:** 10.11604/pamj.2022.43.46.32813

**Published:** 2022-09-28

**Authors:** Issouf Yaméogo, Adama Ouattara, Azize Tiendrébéogo, Jean Claude Romaric Pingdwindé Ouédraogo, Jean Lankoandé

**Affiliations:** 1 District Sanitaire de Dafra, Bobo-Dioulasso, Burkina Faso,; 2 Centre Hospitalier Universitaire Yalgado Ouédraogo, Ouagadougou, Burkina Faso,; 3 Université Joseph Ki-Zerbo, Ouagadougou, Burkina Faso,; 4 Centre Hospitalier Régional de Fada N´Gourma, Fada N´Gourma, Burkina Faso,; 5 Institut de Recherche en Sciences de la Santé, Ouagadougou, Burkina Faso

**Keywords:** Grossesse post-terme, facteurs associés, étude cas-témoins, Post-term pregnancy, factors associated, case-control study

## Abstract

**Introduction:**

la grossesse post-terme constitue une situation à risque pour le fœtus et la mère. Dans notre contexte, peu de données existent sur le sujet, d´où l´intérêt de ce travail qui avait pour objectif d´étudier les facteurs associés à la grossesse post-terme afin de contribuer à une réduction de la morbi-mortalité maternelle et fœtale.

**Méthodes:**

nous avons conduit une étude cas-témoins appariée sur l´âge au département de gynécologie-obstétrique du Centre Hospitalier Universitaire Yalgado Ouédraogo (CHU-YO). Les données ont été collectées entre le 1^er^ janvier 2014 et le 31 août 2014. Les cas étaient les patientes ayant accouché à 41 semaines d´aménorrhée (SA) et plus et les témoins celles ayant accouché entre 37 et 41 SA.

**Résultats:**

l´étude a inclus 153 cas et 153 témoins. Les facteurs significativement associés à la grossesse post-terme étaient le niveau socioéconomique élevé de la mère (odds ratio ajusté (ORa)=3,17; IC95% [1,13; 9,07]), la primiparité (ORa=1,45; IC95% [1,07; 2,51]), et l´antécédent de grossesse post-terme (ORa=7,02; IC95% [2,08; 23,79]).

**Conclusion:**

la reconnaissance précoce des facteurs de risque de la grossesse post-terme aidera le personnel de santé à identifier les femmes dont la grossesse est à haut risque.

## Abstract (en)

**Introduction:**

post-term pregnancy is a risky situation for the fetus and the mother. In our context, few data exist on the subject. This aims to determine the associated factors of post-term pregnancy in order to contribute to a reduction in maternal and fetal morbidity and mortality.

**Methods:**

we conducted a matched case-control study in the department of gynaecology-obstetrics of the Yalgado Ouédraogo University Hospital. Data were collected between 1^st^ January 2014 and 31^st^ August 2014. Cases were patients who delivered at 41 weeks of amenorrhoea and above and controls were those who delivered between 37 and 41 weeks of amenorrhoea.

**Results:**

the study included 153 cases and 153 controls. Risk factors with statistically significant association with post-term pregnancy were high maternal socioeconomic status (adjusted odds ratio (AOR)=3.17; 95% CI [1.13; 9.07]) primiparity (AOR=1.45; 95% CI [1.07; 2.51]), and previous post-term pregnancy (AOR=7.02; 95% CI [2.08; 23.79]).

**Conclusion:**

early recognition of risk factors for post-term pregnancy will help health workers identify women with high-risk pregnancies.

## Introduction

La grossesse post-terme est une situation fréquemment rencontrée en salle d´accouchement. Son incidence est variable d´un contexte à l´autre, et selon la définition adoptée et les méthodes utilisées pour la datation de la grossesse. Selon R. Merger *et al*., la grossesse post-terme se caractérise par une double problématique: d´une part, la future mère est angoissée et pressée d'accoucher, et d´autre part, l´obstétricien redoute une augmentation de la morbi-mortalité maternelle et fœtale [1]. En effet, le risque de mortalité périnatale triple entre 41 semaines d´aménorrhée (SA) et 41 SA + 6 jours et est multiplié par six dès 42 SA [2]. Dans les pays occidentaux, la grossesse post-terme a suscité l´intérêt de beaucoup d´auteurs depuis de nombreuses années [1-3]. Sa prise en charge passe par une bonne surveillance et le déclenchement du travail d´accouchement [1]. En Afrique, peu d´études se sont intéressées au sujet [4-6]. Devant le manque d´équipements médicotechniques adéquats pour sa surveillance et la réanimation des nouveau-nés, il est impératif de mettre l´accent sur la prévention qui passe par la connaissance des facteurs de risques [4]. Cette étude avait pour objectif de déterminer les facteurs associés et les complications maternelles et fœtales de la grossesse post-terme au Burkina Faso, afin de contribuer à la réduction de la morbi-mortalité maternelle et fœtale.

## Méthodes

**Cadre de l´étude:** le département de gynécologie-obstétrique du Centre Hospitalier Universitaire Yalgado Ouédraogo (CHU-YO) nous a servi de cadre pour la conduite de l´étude. Le CHU-YO est le plus grand centre hospitalier du Burkina Faso.

**Type et population d´étude:** il s´est agi d´une étude de type cas-témoins. Les cas étaient les parturientes admises dans le site de l´étude et qui ont accouché à 41 SA ou plus. Les témoins étaient les parturientes admises dans le site de l´étude et ayant accouché entre 37 et 41 SA. Ont été incluses dans l´étude, les parturientes porteuses d´une grossesse unique et ayant bénéficié d´une échographie de datation du premier trimestre. Les parturientes qui avaient une pathologie maternelle grave susceptible de retentir sur le pronostic maternel et fœtal (HTA gravidique, pré-éclampsie, diabète) n´ont pas été incluses dans l´étude. Les données ont été collectées de manière prospective entre le 1^er^ janvier 2014 le 31 août 2014.

**Echantillonnage et taille d´échantillon:** pour déterminer la taille de notre échantillon, nous avons considéré la proportion attendue d´exposées chez les témoins à 5% [2], le rapport de côtes à 4, un niveau de confiance de 95% et la puissance souhaitée pour la détection d'une différence significative entre les deux groupes à 80%. La taille d'échantillon requise pour chaque groupe était alors de 98 et la taille totale minimale de l´échantillon (pour les 2 groupes) était de 196. Les participantes ont été appariées sur l´âge et selon un ratio d´un cas pour un témoin. Quand un cas était identifié, un témoin était aussitôt recherché et enrôlé, consécutivement jusqu´à l´obtention du nombre de participantes attendues.

**Collecte des données:** une fiche a été établie pour collecter les données. Les variables prises en compte dans notre collecte portaient sur: les caractéristiques socio-démographiques maternelles: l´âge, la profession, le statut matrimonial, le niveau socioéconomique, l´ethnie, le lieu de résidence; les antécédents gynéco-obstétricaux de la mère: la régularité du cycle menstruel, l´utilisation de la contraception hormonale, la parité, les antécédents d´accouchement post-terme, les antécédents d´avortement spontané et d´accouchement prématuré; les antécédents chirurgicaux, les antécédents médicaux, le mode de vie; l´âge gestationnel au moment de l´accouchement (calculé sur la base de l´échographie du premier trimestre); les conditions médicales pendant la grossesse: le nombre de consultations prénatales (CPN), la chimioprophylaxie antianémique et antipaludique, le déparasitage, les pathologies pendant la grossesse, les prises d´autres médicaments pendant la grossesse, la réalisation des examens paracliniques (les échographies obstétricales, le Doppler ombilical, l´enregistrement du rythme cardiaque fœtal)); les éléments d´accouchement: le mode d´accouchement, les indications de la césarienne, les complications de l´accouchement par voie basse, les complications du post-partum immédiat.

**Les caractéristiques des nouveau-nés ont été aussi collectées:** l´état du nouveau-né à la naissance, le poids de naissance, le sexe, les signes de post-maturité, les malformations, les lésions traumatiques, la réanimation du nouveau-né, le décès néonatal très précoce. Les données étaient collectées en post-partum immédiat par interview de l´accouchée et à partir des registres des accouchements, les dossiers médicaux et les protocoles postopératoires.

**Analyse des données:** les variables qualitatives ont été résumées sous forme d´effectif et de pourcentage. Les facteurs associés à la grossesse post-terme ont été recherchés à l´aide d´une régression logistique conditionnelle. Les résultats ont été exprimés sous forme d´odds ratios (OR) bruts et ajustés avec leurs intervalles de confiance à 95% (IC 95%) et les p-value associées. Les données ont été analysées à l'aide du logiciel Epi info dans sa version 3.5.3. Les tableaux ont été réalisés à l'aide de Microsoft Office 2010.

**Considérations éthiques:** les fiches de collecte étaient anonymes et seules les patientes qui ont donné leurs consentements éclairés ont été incluses dans l´étude.

## Résultats

### Caractéristiques socio-démographiques et cliniques des patientes

Au total, 153 cas et 153 témoins ont été inclus dans l´étude. La classe d´âge de 25-29 ans était la plus représentée chez les cas et les témoins avec 37,9% des participantes. Les participantes étaient en majorité des femmes au foyer: 32,7% des cas et 34% des témoins. Le niveau socioéconomique était élevé chez 9,8% des cas et 3,27 des témoins. L´ethnie Mossi était l´ethnie majoritaire dans les deux groupes: 60,13% chez les cas versus 66,67% chez les témoins. La majorité des patientes résidaient dans la ville de Ouagadougou: 96,1% des cas et 98,7% des témoins. La parité moyenne dans le groupe post-terme était de 2,10 ± 1,40; elle était de 2,09 ± 1,24 pour les témoins. L´antécédent de grossesse post-terme a été relevé chez 12,58% des cas et chez 1,96% des témoins. Le nombre moyen de CPN était de 3,8 avec des extrêmes de 1 et 7 dans le groupe post-terme et de 3,1 avec des extrêmes de 1 et 8 dans le groupe témoin. Les antipaludiques étaient les médicaments les plus consommés au cours de la grossesse (98,7% des cas versus 95,4% des témoins). Le sexe du fœtus se répartissaient en: masculin (54,9% chez les cas versus 50,98% chez les témoins) et féminin (45,1% chez les cas versus 49,02% chez les témoins). Dans cette étude, 21,56% des nouveau-nés post-termes présentaient des signes de post-maturité représentée entre autres par une peau sèche et fripée, desquamante au niveau des mains et des pieds, et un allongement des phanères. La césarienne était le mode d´accouchement majoritaire chez les cas (55,56%). Chez les témoins, 16,34% ont accouché par césarienne. L´oligoamnios était retrouvé chez 23,08% des cas et chez 4,67% des témoins. La macrosomie était retrouvée chez 10,46% des cas et chez 3,27% des témoins.

### Facteurs associés à la grossesse post-terme

L´analyse des facteurs sociodémographiques, des antécédents gynéco-obstétriques et des conditions médicales associées à la grossesse post-terme sont consignés respectivement dans le Tableau 1, le Tableau 2 et le Tableau 3. Le facteur sociodémographique significativement associé à la grossesse post-terme après une régression logistique conditionnelle multivariée étaient le niveau socioéconomique (ORa=3,17; IC95% [1,113-9,07]). Ainsi, le risque de grossesse post-terme était 3,17 fois plus élevé pour les femmes ayant un NSE élevé comparativement aux femmes ayant un énolase neurospécifique (NSE) bas (p=0,03). Comme antécédents gynécologiques et obstétricaux, la primiparité (ORa=1,45; IC95% [1,107-2,51]) et l´antécédent de grossesse post-terme (ORa=7,02; IC95% [2,08-23,79]) étaient significativement associés à la grossesse post-terme. Il n´y avait pas d´association statistiquement significative entre l´usage des antibiotiques et des antispasmodiques au cours de la grossesse et la survenue de la grossesse post-terme après régression multivariée; les ORa respectifs sont 2,15 (IC95%: 0,02-4,33) et 1,74 (IC95%: 0,88-2,42).

**Tableau 1:** facteurs socio-démographiques associés à la grossesse post-terme, CHUYO, Ouagadougou, 2014

Facteurs	Cas	Témoins	OR brut (IC à 95 %)	P-value	OR ajusté (IC à 95 %)	P-value
	n (%)	n (%)				
**Statut matrimonial**						
Mariée	100 (65,4)	113 (73,9)	0,66 (0,40-1,09)	NS*		
Concubinage	49 (32)	34 (22,2)	1,64 (0,98-2,74)	NS		
Célibataire	4 (2,6)	6 (3,9)	1			
**Profession**						
Femme au foyer	50 (32,7)	52 (34)	0,94 (0,58-1,51)	NS		
Secteur informel	30 (19,6)	29 (19)	1,04 (0,59-1,84)	NS		
Salariée	40 (26,1)	45 (29,4)	1,84 (1,51-2,40)	0,04	1,26 (0,75-1,83)	NS
Elève/étudiante	33 (21,6)	27 (17,6)	1			
**NSE**						
NSE bas	96 (62,75)	97 (63,40)	1			
NSE moyen	42 (27,5)	51 (33,3)	0,75 (0,46-1,23)	NS		
NSE élevé	15 (9,8)	5 (3,27)	3,21 (1,13-9,08)	0,03	3,17 (1,13-9,07)	0,03
**Ethnie**						
Bissa	12 (7,84)	10 (6,54)	1			
Dioula	15 (9,8)	12 (7,84)	1,27 (0,57-2,82)	NS		
Gourounsi	17 (11,11)	20 (13,07)	0,83 (0,41-1,65)	NS		
Mossi	92 (60,13)	102 (66,67)	0,75 (0,47-1,20)	NS		
Peulh	10 (6,53)	7 (4,58)	1,45 (0,54-3,93)	NS		
Autres*	7 (4,58)	2 (1,31)	3,61 (0,73-17,71)	NS		
**Provenance**						
Ouagadougou	147 (96,1)	151 (98,7)	0,32 (0,61-1,63)	NS		
Province	6 (3,9)	2 (1,3)	1			

NS*: non significatif

**Tableau 2:** antécédents gynéco-obstétricaux associés à la grossesse post-terme, CHUYO, Ouagadougou, 2014

Facteurs	Cas	Témoins	OR brut (IC à 95 %)	P-value	OR ajusté (IC à 95 %)	P-value
	n (%)	n (%)				
**Cycle menstruel**						
Régulier	112 (73,2)	104 (67,97)	1			
Irrégulier	41 (26,8)	49 (32,03)	0,77 (0,47-1,27)	NS		
**Contraception**						
Contraception (+)	46 (30,1)	47 (30,7)	1			
Contraception (-)	107 (69,9)	106 (69,3)	1,03 (0,63-1,67)	NS		
**Parité**						
Primipare	75 (49,02)	57 (37,25)	1,61 (1,02-2,55)	0,04	1,45 (1,07-2,51)	0,04
Paucipare	57 (37,25)	76 (49,67)	0,60 (0,38-2,94)	NS		
Multipare	21 (13,07)	20 (13,73)	1			
**Grossesse post-terme**						
Antécédent (+)	19 (12,42)	3 (1,96)	7,08 (2,05-24,49)	0,0005	7,02 (2,08-23,79)	0,00000
Antécédent (-)	134 (87,58)	150 (98,04)	1			
**Avortement spontané**						
Antécédent (+)	23 (15)	27 (17,6)	0,82 (0,44-1,55)	NS		
Antécédent (-)	130 (85%)	126 (82,34%)	1			

**Tableau 3:** conditions médicales associées à la grossesse post-terme, CHUYO, Ouagadougou, 2014

Conditions médicales	Cas	Témoins	OR brut (IC à 95 %)	P-value	OR ajusté (IC à 95 %)	P-value
	n (%)	n (%)				
**CPN**						
CPN≥3	149 (97,4%)	145 (94,8%)	1,48 (1,14-3,65)	0,02	1,98 (1,51-3,63)	0,04
CPN<3	4 (6%)	8 (5,2%)	1			
**Fer + Acide folique**						
Oui	150 (98%)	150 (98%)	1 (0,19-5,03)	NS		
Non	3 (2%)	3 (2%)	1			
**Sulfadoxine + Pyriméthamine**						
Oui	151 (98,7%)	14 6(95,4%)	3,61 (0,73-17,71)	NS		
Non	2 (1,3%)	7 (4,6%)	1			
**Albendazole**						
Oui	26 (17%)	24 (15,7%)	1,10 (0,60-2,01)	NS		
Non	127 (83%)	129 (84,3%)	1			
**Antibiotiques**						
Oui	21 (13,7%)	8 (5,2%)	2,88 (1,23-6,73)	0,01	2,15 (0,02-4,33)	NS
Non	132 (86,3%)	145 (94,8%)	1			
**Antispasmodiques**						
Oui	62 (40,5%)	50 (32,7%)	2,40 (1,87-3,23)	0,03	1,74 (0,88-2,42)	NS
Non	91 (59,5%)	103 (67,3%)	1			
**Médicaments antipaludiques**						
Oui	151 (98,7%)	146 (95,4%)	3,61 (0,73-17,71)	NS		
Non	2 (1,3%)	7 (4,6%)	1			

## Discussion

De cette étude, il ressort que le niveau socio-économique élevé, la primiparité et l´antécédent de grossesse post-terme étaient les facteurs influençant la survenue de la grossesse post-terme. Par contre, la prise de médicaments pendant la grossesse et le sexe du fœtus n´étaient pas associés à la grossesse post-terme.

Le niveau socio-économique élevé était un facteur indépendant associé à la grossesse post-terme (ORa=3,17; IC95% [1,13-9,07]. Cela pourrait s´expliquer par le fait que les patientes qui ont un niveau socioéconomique élevé aient une activité physique réduite (sédentarité, présence d´une aide-ménagère, etc.). En effet, un niveau socio-économique élevé favoriserait le prolongement de la grossesse du fait d´une meilleure observation du repos. A contrario, plus les conditions socio-économiques sont défavorables, plus la gestante court le risque d´accouchement prématuré [7].

La parité était un facteur associé à la grossesse post-terme dans cette étude. La primiparité était un facteur significativement associé à la grossesse post-terme (OR=1,45; IC95% [1,07-2,51]). Nos résultats sont similaires à ceux de Mittendorf *et al*., Smith *et al*. qui ont démontré que les multipares accouchent en moyenne bien avant les primipares [8,9]. Cependant, ce facteur semble controversé et plusieurs auteurs ne citent aucune relation entre la parité et la durée de la grossesse [10,11]. Beischer *et al*. dans une étude prospective portant sur 2972 patientes suivies depuis le premier trimestre n´a mis en évidence de différence significative entre primiparité et multiparité [11]. Ces discordances pourraient s´expliquer par une différence dans la définition de la grossesse post-terme.

Les parturientes ayant un antécédent de grossesse post-terme avaient un risque 7 fois plus élevé de voir leur grossesse se prolonger par rapport à celles sans antécédent de grossesse post-terme (ORa=7,02; IC95% [2,08-23,79]). Dans la littérature, l´existence d´un antécédent de grossesse post-terme serait un facteur important dans la survenue d´une autre grossesse post-terme. Pour Vorherr *et al*., une femme ayant un antécédent de grossesse post-terme à 50% de risque de réitérer [12]. Mogren *et al*. ont montré que le risque relatif pour la grossesse de se prolonger après une grossesse post-terme augmenterait de 2 à 3 fois [13]. La mesure d´association dans notre série (ORa=7,02; IC95% [2,08-23,79]) semble plus importante et pourrait s´expliquer par le fait que le diagnostic de la majorité des grossesses post-terme dans les antécédents des patientes se serait basé sur la DDR, ce qui a eu pour inconvénient de surestimer la proportion des patientes ayant un antécédent de grossesse post-terme.

Le sexe du fœtus n´était pas associé à la grossesse post-terme (ORa=1,17; IC95% [0,74-1,83]). Cela concorde avec les résultats des études de Smith *et al*. qui trouvaient une durée identique (283 jours) pour chaque sexe à partir d´une étude qui porté sur 1514 grossesses [9]. Cependant, pour certains, les garçons entraineraient une durée de gestation plus longue. En effet, Vorherr *et al*., et Divon *et al*. ont rapporté une prédominance du sexe masculin dans les grossesses post-terme [12,14]. Selon lesdites études, la durée moyenne de la grossesse était plus élevée chez les garçons (290,6 ± 8,9 jours) par rapport à celle des filles (279,8 ± 8,6 jours). Toutefois, les raisons pour lesquelles le sexe masculin exposerait plus à la grossesse post-terme restent encore mal élucidées [14].

La prise de médicaments pendant la grossesse n´était pas associée à la grossesse post-terme. Cependant, Stewart *et al*. avait noté un lien entre la prise de médicaments pendant la grossesse et le prolongement de celle-ci [15]. Néanmoins, il est utile de rappeler, ici, que l´automédication abusive au cours de la grossesse peut comporter des effets délétères pour le fœtus.

**Limites:** l´étude pourrait présenter une certaine limite, notamment l'origine hospitalière des données et le fait que l´étude s´est déroulée dans un seul hôpital, fut-il de référence et le plus grand favorise un biais de sélection.

## Conclusion

La grossesse post-terme demeure une préoccupation en salle d´accouchement. Certains de ses facteurs associés décrits dans la littérature ont été vérifiés dans notre série. La grossesse post-terme a été associée à une augmentation de la morbidité maternelle et fœtale en comparaison avec les accouchements à terme. La morbidité maternelle a été essentiellement dominée par la césarienne. La mortalité périnatale n´était pas différente entre les nouveau-nés issus des accouchements en post-terme et ceux issus des accouchements à terme. La reconnaissance précoce des facteurs de risque de la grossesse post-terme aidera le personnel de santé à identifier les femmes dont la grossesse est à haut risque. Après cette analyse des facteurs associés de la grossesse post-terme, la mise en route d´un plaidoyer en vue de l´acquisition de moyens de surveillance et des moyens de déclenchement du travail devrait s´organiser pour le bien-être de la mère et de l´enfant.

### Etat des connaissances sur le sujet

- L´existence d´un antécédent de grossesse post-terme est un facteur important dans la survenue d´une autre grossesse post-terme;
- La césarienne est le principal mode d´accouchement lors de la grossesse post-terme.

### Contribution de notre étude à la connaissance

- Les facteurs de risque de la grossesse post-terme dans le contexte du Burkina Faso sont le niveau socio-économique élevé de la patiente, la primiparité et l´antécédent de grossesse post-terme.
